# Spotlight on early-career researchers: an interview with Andrea Henle

**DOI:** 10.1038/s42003-019-0285-x

**Published:** 2019-02-07

## Abstract

Dr. Andrea Henle is an Assistant Professor of biology at Carthage College in Wisconsin. Her research focuses on uveal melanoma, using zebrafish as a model system, and spans molecular and cellular mechanisms of cancer progression, immunology, and even space biology. As part of our series on early-career researchers, we asked Dr. Henle to talk to us about her research and her passion for teaching undergraduate students. Dr. Henle also has some advice for young scientists pursuing an academic career, which we think is equally valuable for anyone starting out on their unique career path.

**Figure:**
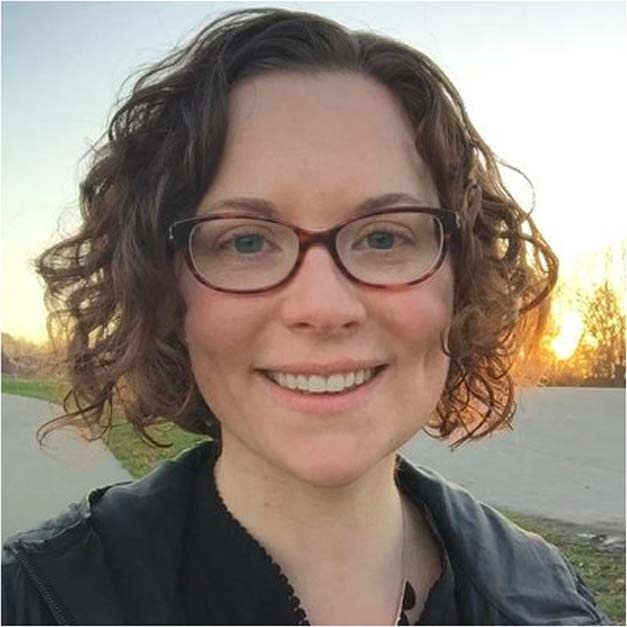
Image credit: Andrea Henle

Please tell us about your research interests.

I am interested in understanding the molecular pathways that lead to the progression of uveal melanoma. Uveal melanoma is the most common eye cancer in the U.S. We currently have successful treatments for the cancer when it is localized in the eye, however, we do not have therapies for the disease when it metastasizes to other sites, such as the liver. If we can gain a better understanding of molecular signals and genes that might cause the cancer to become metastatic or recur years later, then we might be able to develop more effective, targeted therapies for this deadlier form of the disease. I am currently using a zebrafish model of melanoma to study these processes.

I am also interested in space biology and studying the effect that factors during spaceflight (such as radiation and microgravity) might have on cancer development during long duration space missions. Space biology is a very exciting and interdisciplinary field, and I am looking forward to using my established zebrafish model to contribute to our understanding of cancer under simulated space conditions.

What has your journey been to this point?

As an u;ndergraduate student at a small liberal arts college, I had a fairly strong inclination that I wanted to combine a career in research and teaching, similar to the professors at my institution. I spent 3 years as a teaching assistant for the introductory biology laboratories at my college, where I discovered how much I enjoyed helping other students learn the material and ask biological questions. I also had the opportunity to conduct research in physiology during one summer at my campus and in cellular immunology the following summer through the SURF program at Mayo Clinic. The combination of these research and teaching experiences helped me realize that I would enjoy pursuing a graduate degree in immunology at Mayo Clinic and contributing to the field of cancer immunology. I remained equally passionate about both teaching and research, so I always made it a point to dedicate time to improving my skills in both areas during my training. While at Mayo, I continued to pursue teaching opportunities outside of the research laboratory, through being a TA for graduate school courses, or an adjunct instructor in the evening at the local community college and local liberal arts college. These experiences ultimately helped me be successful as a scientist and a teaching professor. After graduating with my Ph.D., I applied to several combined teaching and research postdoctoral programs. I ended up selecting a joint teaching and research postdoctoral fellowship between MIT and Singapore University of Technology and Design. This innovative program allowed me to conduct cutting edge cancer research at MIT and teach MIT’s biology curriculum for one semester in Singapore. It was a very exciting partnership that allowed me to work with scientists from all over the world, establish a new model system (zebrafish) to study uveal melanoma, and learn how to teach in a multicultural educational setting. After my postdoctoral fellowship, I spent a year as a Visiting Assistant Professor at Bard College before accepting my current position as an Assistant Professor of biology at Carthage College.

What are your predictions for your field in the near future?

I believe therapies for diseases such as uveal melanoma will become more targeted and personalized in the next few years. We will continue to make advances in our understanding of the basic biology of cancer cells, which will allow us to better determine the molecular markers associated with a patient’s tumor and select the appropriate drug or treatment based on that information. I am also very excited for the field of cancer immunotherapy and the new advances that are being made to use cells of the immune system and/or antibody-based therapeutics to target different types of tumor cells.

Can you speak of any challenges that you have overcome?

I think some of the biggest challenges I had to overcome dealt with the two-body problem. My husband is also a scientist, so along the way we had to determine how to support both of our careers and maintain our family/personal life. It was not always easy to know which choice was the right one to make. At times, we had to make sacrifices such as living apart temporarily for new career opportunities. In the end, we somehow made it work and I am happy to report we both have tenure-track positions now and no foreseeable plans to live apart long-distance. I feel for any scientists facing these challenges right now. Unfortunately, since every situation is different and the job market offers varied opportunities each year, the only advice I can provide is to maintain clear communication in your relationship regarding your shared goals and values.

What advice would you give to your younger self?

Continue to network! I know that networking doesn’t always come naturally to everyone, but there were many times in my career path when I received help and guidance by reaching out to an alum of my current institution or program, a collaborator of a colleague, or someone I may have recently became acquainted with at a conference or workshop. Often times I initiated discussions with others to either brainstorm new project ideas, to offer help with a project the other person might be leading, or to inquire about that person’s career path. Some of these discussions ultimately led to me being offered a chance to teach at their institution, to submit a grant proposal with their team, or to work collaboratively on developing materials for a new course. Never underestimate the power of networking. And when you are in a position to mentor others who are just starting their career, make sure to pay it forward.

Bonus question: What is one underappreciated thing you wish everyone knew about zebrafish?

Zebrafish are actually a great model organism to use to study cancer as their genomes are relatively similar to humans and their transparency early during development allows for easy tracking and monitoring of fluorescent or pigmented tumor cells. There are even adult zebrafish that are transparent, so cancer development and progression can be monitored over a longer period of time.

*This interview was conducted by Chief Editor Brooke LaFlamme*

